# Exploring PAH kinetics in wild vs. transplanted triploid and diploid oysters at a contaminated field site using immunological techniques

**DOI:** 10.1007/s10661-023-12064-1

**Published:** 2023-11-13

**Authors:** Kristen M. Prossner, Ellen Harvey, Michael A. Unger

**Affiliations:** Virginia Institute of Marine Science, William & Mary, P.O. Box 1346, Gloucester Point, VA 23062 USA

**Keywords:** Bivalves, Biomonitoring, Polycyclic aromatic hydrocarbons, Biosensors, Immunofluorescence, Aquaculture

## Abstract

*Crassostrea virginica* is a well-established bivalve species for biomonitoring persistent organic pollutants such as polycyclic aromatic hydrocarbons (PAH) in aquatic environments. Differing biomonitoring methods employing either wild oysters inhabiting sites of interest or naïve cultured oysters deployed to sites for extended periods can be used for site evaluations. However, important differences in total contaminant concentrations accumulated have been observed between the wild and transplanted groups. Furthermore, although rearing cultured triploid oysters is widely popular in commercial farming, the difference in contaminant bioaccumulation potential between triploid and diploid cultured oysters is vastly understudied, particularly for organic contaminants such as PAH. This study explores differences in PAH kinetics between transplanted triploid and diploid cultured oysters and wild oysters at a PAH-impacted site during a 6-week field exposure study using novel immunological techniques: antibody-based biosensor technology and immunofluorescence visualization. Conventional chemical analysis of oyster tissue was also conducted for comparison. While differences were observed in the oyster interstitial fluid between the wild and transplanted oysters throughout the study, whole tissue analysis revealed differing trends at each time point. Our findings suggest that insufficient equilibration time may contribute to the differences observed between groups. Furthermore, when combined with visual evidence via immunofluorescence, internal partitioning of contaminants may be an important determinant for total concentrations measured. A better understanding of the differences observed between wild and transplanted oyster groups is necessary for improved biomonitoring. Our study highlights the value in employing novel immunological techniques to explore possible mechanisms driving these differences.

**Supplementary Information:**

The online version contains supplementary material available at 10.1007/s10661-023-12064-1.

## Introduction

As sessile, benthic filter-feeders, bivalves such as *Crassostrea virginica* are susceptible to the accumulation of lipophilic contaminants such as polycyclic aromatic hydrocarbons (PAH) and have limited capacity for biotransformation of these compounds (James, 1989). Accordingly, *C. virginica* are a well-established biomonitoring species for persistent organic pollutants such as PAHs. They have served as key sentinel species to determine the bioavailability of PAHs in aquatic ecosystems in large-scale monitoring programs such as NOAA Mussel Watch since its establishment in 1976 and global programs such as the UNESCO-IOC International Mussel Watch Project (Farrington et al., 1983, 2016; Goldberg et al., 1978; Wade et al., 1998). Two methods for biomonitoring exist. Passive biomonitoring involves the collection of wild bivalves inhabiting pollution monitoring sites and has been historically employed in long-term monitoring efforts such as NOAA Mussel Watch (Farrington et al., 1983; Sericano et al., 1995). Furthermore, they can be immediately sampled since it can be assumed that wild oysters have equilibrated with the environment. Active biomonitoring involves the deployment of caged bivalves, either collected from a pristine site and relocated or purchased from an aquaculture. This method can overcome the heterogenous distribution of wild bivalves or complete absence of bivalves in sites of interest (Lacroix et al., 2015). The stock, origin, and biological parameters of the species used in active biomonitoring can be more easily controlled, reducing variability and enhancing replication of the study (Besse et al., 2012). However, bivalves must be deployed at sites of interest for an extended time to reach steady state with the environment (Sericano et al., 1996). Furthermore, the reported optimal length of time required for equilibration is highly variable, ranging from 3 weeks to up to 2 years (Bodin et al., 2004; Marigomez et al., 2013; Beyer et al., 2017).

Although both techniques are employed, differences in total concentrations have been observed in PAH uptake and depuration when two mussel populations are compared (Kazour & Amara, 2020). In a cross-transplantation study, Sericano et al. (1996) found that on the final day of a 48-day uptake phase, *C. virginica* oysters transplanted from a pristine to an impacted site in the Houston Ship Channel accumulated PAHs to levels comparable to those detected in oysters native to the impacted site. On the final day of a 50-day depuration period at the pristine site, the transplanted oyster (returned to their original location) concentrations were lower than concentrations measured in oysters native to the PAH-impacted site (transplanted to the pristine site). The authors proposed that newly exposed oysters can eliminate contaminants at a much faster rate than those chronically exposed. In a 6-month PAH accumulation study, Schøyen et al. (2017) found that transplanted *M. edulis* mussels never accumulated PAHs to levels observed in the native population at an impacted site. Lacroix et al. (2015) found that transplanted *Mytilus* spp. mussels accumulated higher levels of PAH in the digestive gland relative to that of native oysters at an impacted site in the Brest Harbor (France). Not only are there inconsistencies in which group (native relative to transplant) assimilates the highest PAH concentrations, but there is also a lack of consensus as to why these differences are observed. Some studies attribute differences between groups as being driven by chemical kinetic rates and lack of sufficient time for reaching steady state (Greenfield et al., 2014; Schøyen et al., 2017; Sericano et al., 1996). Others suggest that biological response drives the observed differences and that chronically exposed bivalves native to contaminated sites may have set up metabolic adaptations to reduce stress response (Faria et al., 2010; Marigomez et al., 2013; Lacroix et al., 2015). Therefore, the questions of why differences are observed, and which strategy is a more appropriate evaluation of changes in environmental quality due to contamination warrant further scrutiny to enhance the precision of future biomonitoring efforts. Addressing these differences is especially important when these approaches are employed for the assessment of potential risk to human health against safety thresholds (Beyer et al., 2017).

Oysters for active biomonitoring can be sourced from pristine sites or purchased from aquaculture farms (Beyer et al., 2017). There has been an increasing incentive for farms to produce triploid oysters due to their faster growth rates and ability to reach market-size earlier than diploid counterparts. This results in maintaining a desirable meat quality year-round for consumers (Cheney, 2010; Nell, 2002). In the Chesapeake Bay (USA), triploid *C. virginica* have been widely adopted in aquaculture and account for most oysters brought to market (Callam et al., 2016; Wadsworth et al., 2019). The commercial advantages are attributed to energy reallocation from gametogenesis to somatic growth seen in triploids (Allen & Downing, 1986). Triploid oysters are partially sterile, induced through chemical or physical methods of manipulation (Stanley et al., 1984; Allen et al., 1989; Guo et al., 1996; Nell, 2002). Therefore, spawning and subsequent emaciation due to depleted energy reserves does not occur to the same extent as seen in diploid counterparts (Nell, 2002). Multiple studies have assessed the effect of ploidy as it relates to oyster health and performance in commercial aquaculture production, such as disease susceptibility (Barber & Mann, 1991; Dégremont et al., 2015; Meyers et al., 1991) and growth (Garnier‐Géré et al., 2002; Walton et al., 2013). However, the effect of contaminant stress as well as the differences in chemical kinetics in triploids relative to diploids exposed to environmental contaminants such as PAH is vastly understudied. Studies evaluating the bioaccumulation differences in triploids and diploids for environmental contaminants are generally scarce. Only one other study has been documented examining the effect of ploidy on PAH accumulation (Miles et al., 2014). To date, the difference in PAH uptake and depuration in transplanted triploid and transplanted diploid oysters relative to wild oysters inhabiting impacted sites has not been investigated. As a popular commercial product, the increased growth rate of triploid oyster strains necessitates further investigation into PAH bioaccumulation potential.

Antibody-based biosensor technology, specifically the KinExA Inline Sensor coupled with a monoclonal antibody uniformly selective for 3–5 ring PAHs, mAb 2G8, has previously demonstrated its value as a near real-time and low-cost quantitative screening technique for environmental monitoring of PAH contamination (Camargo et al., 2022; Conder et al., 2021; Hartzell et al., 2017; Li et al., 2016; Prossner et al., 2022). The antibody mAb 2G8, covalently bonded to a fluorescent tag, AlexaFluor 647 (AF647), has also been previously employed in immunohistochemical (IHC) investigation of the accumulation of PAH mixtures in specific oyster tissues (Prossner et al., 2023) demonstrating that the intensity of the mAb signal in oyster tissues is proportional to the PAH exposure history in the laboratory or field.

In this study, we explore the difference in PAH concentration between wild and transplanted triploid and diploid oysters at the start and end of a 30-day uptake phase and at the end of a 2-week depuration phase. We compare PAH concentrations in the oyster interstitial fluid (aqueous phase) measured by biosensor to concentrations in the tissue phase measured by GC–MS. Furthermore, we investigated differences in internal PAH partitioning between wild oysters and transplanted triploid oysters, specifically the differences in partitioning between the digestive gland (i.e., hepatopancreas) and gill, to gain insight into possible mechanisms driving the observed differences in biomonitoring approaches. The uptake phase took place at a known PAH-contaminated field site, the historic location of the Republic creosote plant in the Elizabeth River (Virginia, USA). Oysters were then relocated to the York River (Virginia, USA) for a 14-day depuration period. This is the first-documented study comparing these three populations in a PAH exposure study.

## Methods and materials

### Experimental design

**Fig. 1:**
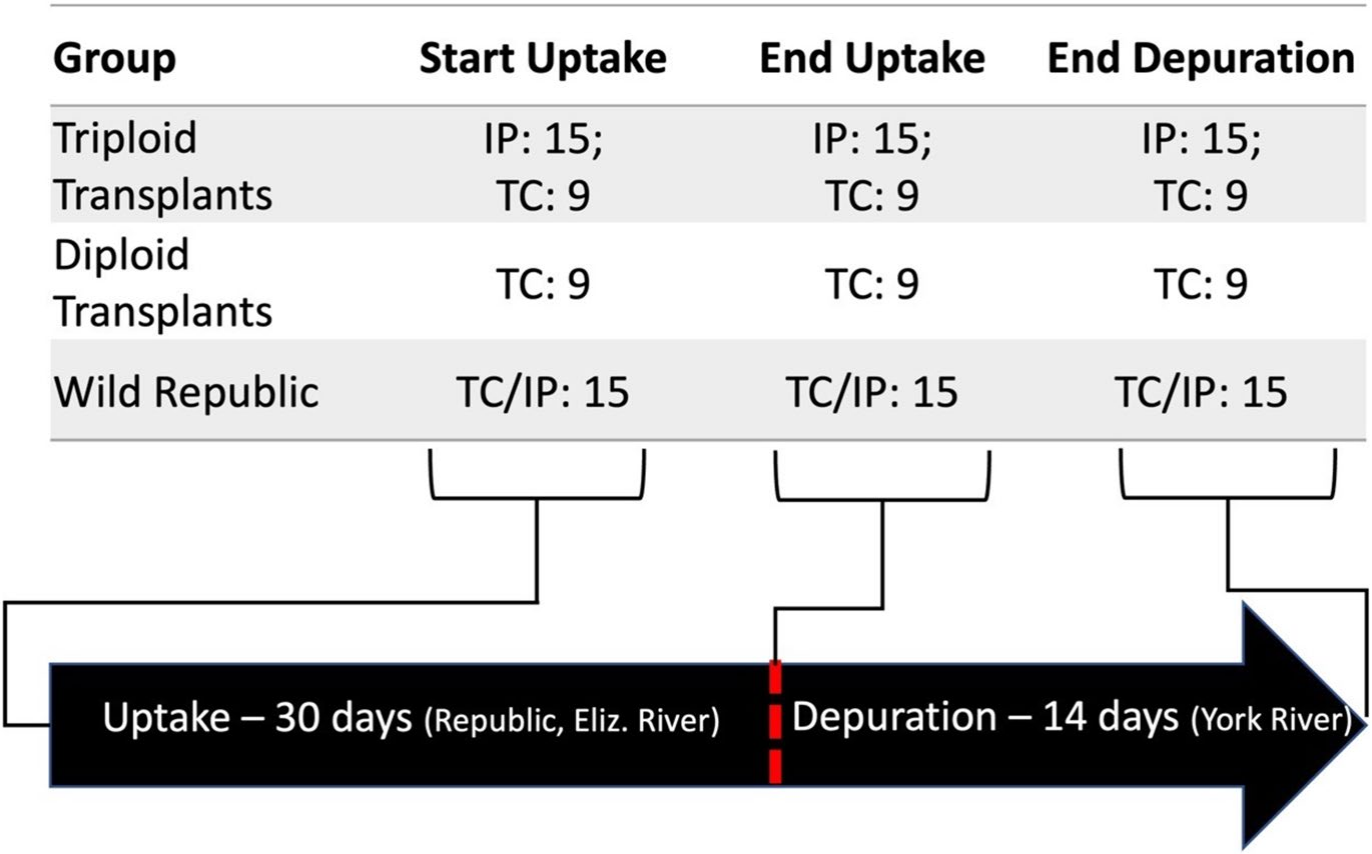
Sampling regime for each oyster group at each time point throughout the 44-day experiment. IP, number of oysters collected for analysis of digestive gland fluid and gill fluid to evaluate internal partitioning. TC, number of oysters collected for analysis of oyster interstitial fluid to compare mean PAH concentrations across oyster groups. IP/TC, samples collected for both analyses of respective objectives that were conducted on the same set of oysters. Number of samples collected at each time point (15 or 9) depicts the total number of oysters collected across 3 cages (i.e., 5 oysters per cage or 3 oysters per cage for each time point). Not depicted in schematic: a gill and digestive gland tissue fragment was collected from one IP oyster per group per time for immunohistochemistry (IHC)

The uptake phase of the study took place in spring–summer conditions (late May to early July) at the Republic field site in the Elizabeth River (Virginia, USA), a sub-tributary located near the mouth of the Chesapeake Bay. Republic is the site of a former creosote wood treatment plant and known PAH hotspot (Di Giulio & Clark, 2015; Prossner et al., 2022). Oysters were held in cages secured to a dolphin piling centrally located at the site in subtidal conditions. The sampling regime throughout the experiment is described in Fig. 1. The uptake phase lasted for a 30-day period. On the final day of uptake, oysters were relocated to the York River at Gloucester Point, VA, to depurate for 14 days. Oyster cages were secured to concrete pier pilings and held in subtidal conditions. Oyster were sampled at the start of uptake (T0), end of uptake (T4), and at the end of depuration (T6). Three replicate cages, each containing one bag of wild oysters and diploid oysters and two bags of triploid oysters (one bag for total concentration comparison and internal partitioning, respectively), were employed to avoid overcrowding of oysters and for protection against predation and loss due to vessel traffic and large wakes.

Wild oysters were collected from pilings and shoreline at the Republic site as samples of convenience. Farmed diploid oysters were reared in the Rappahannock River from seed in Middlesex County, VA, USA (~ 112 km from study site). Farmed triploid oysters were reared in Gloucester County, VA, USA, in the York River (~ 80 km from study site). Oyster shell heights from the umbo to the bill edge were measured using calipers prior to experimentation.

### Sample preparation for biosensor analysis

For biosensor analysis of transplanted triploid and diploid oysters total PAH concentrations, whole (i.e., in-shell) oysters were collected, immediately placed in plastic bags, and transported in coolers on ice to the laboratory (~ 65 km away). Three individual oysters were sampled per group per time point. Upon arrival, the animals were immediately stored whole at − 20 °C until further preparation. For processing, oysters were thawed, opened with a shucking knife, and an aliquot of oyster interstitial fluid pooled within the shell was collected using a disposable glass Pasteur pipet. The aliquot of fluid was transferred to a glass 20-mL scintillation vial. The oyster interstitial fluid was then filtered through a 0.45-µm PTFE syringe filter, the filtrate transferred to a clean scintillation vial, and stored at − 20 °C until analysis. Due to constraints on the availability of diploid oysters for transplantation, the total concentration was the sole measurement collected for this group by biosensor. Remaining soft tissue of the three individual oysters per group per cage at each time point was pooled (i.e., triplicate pooled samples per group per time point) and transferred to a glass screw-top jar for further preparation for GC–MS analysis.

For biosensor analysis of internal PAH partitioning in triploids, sample collection, transport from the field, and storage prior to preparation were described above. Separate groups of triploid oysters were used for the comparisons of total concentrations and internal partitioning to ensure adequate sample volumes. The gill and digestive gland were dissected from 5 individual oysters and pooled. Based on a preliminary experiment, a minimum of 5 oysters is required to achieve sufficient volume for biosensor analysis (~ 1 mL). A small section of each tissue type from one of the 5 oysters per group per cage was collected for further cryopreservation and IHC preparation described in the below section. The pooled samples were homogenized via manual maceration to create one homogenized gill sample and one homogenized digestive gland sample. One sample of each tissue was collected per group per cage at each time point (i.e., the gill and digestive gland was removed from 5 individual oysters per group per time point for 3 replicate cages). Homogenized gill and digestive gland samples were then centrifuged, and the supernatant was collected using a glass disposable Pasteur pipet and transferred to a glass scintillation vial. Gill and digestive gland fluid was then filtered through a 0.45-µm PTFE syringe filter and transferred to a clean scintillation vial. Samples were stored at − 20 °C until analysis.

Sample preparation for wild oysters proceeded as described above; however, due to the constraint of limited abundance of wild oysters at the Republic site, oyster interstitial fluid for total concentration comparison, gill and digestive gland fluid for internal partitioning, and individual tissue sections for IHC were collected from the same individual oysters. Therefore, an aliquot of oyster interstitial fluid from 5 oysters per cage per time point were sampled for total concentration comparison. Then, respective gill and digestive glands were dissected, pooled, and homogenized to generate digestive gland fluid and gill fluid. The remaining tissue following centrifugation was reserved and combined with the remaining soft tissue of the 5 oysters per cage per time point. The soft tissue, including residual gill and digestive gland tissue post-homogenization, was then pooled to generate triplicate samples per time point for further preparation for GC–MS analysis. This constraint led to an unequal sample size between groups so that the minimum sample size requirement for generating gill and digestive gland fluid could also be achieved. This was accounted for in subsequent statistical analyses, as described in the corresponding section.

### Biosensor analysis

The procedure for analysis with the KinExA Inline Sensor (Sapidyne Instruments) as well as the selection and screening of mAb 2G8 has been described previously (Li et al., 2016; Prossner et al., 2022; Spier et al., 2011, 2012). Briefly, the sensor functions as a kinetic assay in which AF647-tagged mAb 2G8 mixes with an aqueous sample and binds PAH. The sample-antibody solution is passed over antigen (1-pyrene-butyric acid), held stationary in a glass flow cell in front of the detector, and remaining free antibody will bind to the antigen and a signal response (dV) is measured. Prior to sample analysis, a 5-point calibration curve is generated using series of phenanthrene standards (0.5 to 2.5 µg/L) and laboratory blank (double deionized water; ddH2O) to establish the linear range of the detector’s response. To stay within the range of the calibration curve, samples are diluted with ddH20, if necessary. The measured signal response and required dilution factor are then used to calculate a total 3–5 ring PAH concentration (µg/L).

### GC–MS analysis

Preparation of oyster tissue for GC–MS analysis has been described previously (Prossner et al., 2022). Composite tissue samples were thawed and homogenized using a Virtis Homogenizer. All components for homogenization (stainless steel blades, glass reservoir, and lid) were cleaned prior to each sample. The liquified sample was then poured into a pre-cleaned Pyrex glass dish and freeze-dried along with a laboratory blank. Samples and blanks were spiked with a deuterated PAH surrogate standard and solvent extracted using an accelerated solvent extractor. Samples were then reduced under a gentle stream of nitrogen held in a 40 °C bath in a TurboVap evaporator. For size exclusion separation, a standard protocol was followed using a high-performance liquid chromatograph (HPLC) coupled with a gel permeation column (GPC). Following HPLC-GPC, the remaining 2.0 mL of sample extract was reserved for lipid content analysis. The residual extraction solvent was then allowed to evaporate from the sample extract at ambient room temperature and the difference in mass between the dried sample residue and the full sample was determined to estimate lipid content. Total extractable lipid content was then calculated as a percentage of tissue dry weight. Sample fractionation and polar compound removal were achieved via open column chromatography containing deactivated silica gel through a series of elutions. The tissue extracts were then solvent exchanged to 100% dichloromethane and concentrated to a final volume. A set of calibration standards was spiked with an internal standard, p-terphenyl, and used to generate a calibration curve for analysis. Both parent and methylated compounds were analyzed for a total of 64 analytes. Concentrations are reported in µg/kg dry weight.

### Tissue specimen preparation and IHC processing for imaging

Section preparation and IHC processing for oyster tissue have been described previously (Prossner et al., 2023). Gill and digestive gland tissue fragments (~ 6 mm^3^) were dissected from each oyster and embedded in optimal cutting temperature (OCT) media in a disposable cryo-mold. Approximately 300 mL of pentane was held in a small stainless-steel bowl within a bath of liquid nitrogen. The mold was then partially submerged in the cooled pentane until entirely frozen. Frozen blocks were stored at − 80 °C. Twenty-four hours prior to cryosectioning, blocks were warmed to − 20 °C. Upon cryosectioning, blocks were removed from their mold and frozen to an aluminum chuck and 10-µm sections were cut at − 10 °C on a cryostat (Leica CM3050 S). Sections were pressed onto a positively charged glass slide (Superfrost Plus; Fisher Scientific) at room temperature. Once mounted, sections were fixed in 4% paraformaldehyde in 1X phosphate buffered saline (PBS) for 20 min, then immersed in a blocking and permeabilization solution for a 1-h incubation period. Sections were then incubated in an AF647-tagged mAb 2G8 coating solution overnight in a moist chamber at 4 °C. The next day, sections were incubated with 4′,6-diamidino-2-phenylindole (DAPI) in 1X PBS for 15 min. Between each step, sections were thoroughly washed via three 5-min baths in 1X PBS. Upon carefully wiping off excess moisture, a coverslip was mounted using Dako mounting media. After drying, the edges around the cover slip were sealed with clear nail varnish. Preparation of negative controls followed the same procedure but was incubated in a blank antibody solution overnight. Images were captured using a FLUOVIEW FV1200 confocal laser scanning microscope (Olympus) with filter sets for DAPI and AF647.

### Image analysis

The procedure for image analysis has been described previously (Prossner et al., 2023). Integrated density was measured using FIJI image analysis software (Schindelin et al., 2012). Integrated density is the product of total selected measurement area and mean gray value within the area. Upon analysis, a uniform scale and intensity threshold was set across images for comparison. In gill tissue, measurements in triplicate were made on three plicae per image. For subsequent comparison between groups, individual Welch two-sample *t*-tests were performed at each time point to determine if a significant difference in group means was present.

### Statistical analyses

The assumptions of homoscedasticity and normality were not met to conduct an analysis of variance (ANOVA), based on results on a Shapiro–Wilk test for normality, Bartlett’s test for homogeneity of variance, and plotting of residuals. Therefore, the response factor, PAH concentration (µg/L), was log_10_-transformed to better meet or approximate these assumptions. In addition, the experimental set-up did not allow for the assumption of independent data to be met when means are compared across all time points. Because different individual oysters were sampled at each time point, a repeated measures ANOVA is inappropriate. Also, comparing the rate of change in PAH concentration over time between groups was not a research objective. Therefore, separate ANOVAs were conducted at each time point to meet all assumptions for ANOVA which was sufficient for testing our hypotheses. For determining a significance difference in mean total PAH concentrations between groups, a one-way ANOVA was conducted in R using the pipeline-friendly “rstatix” package for streamlined incorporation of statistical results into figures when data were plotted using package “ggplot2.” If results of the ANOVA indicated that the true difference between means was not equal to zero, a Games-Howell post hoc test was used to account for unequal sample size. For determining significant difference in mean gill fluid and digestive gland fluid between groups, individual two-way ANOVAs were conducted at each time point. A 0.05 significance level was selected for all analyses.

## Results and discussion

### Total concentration comparison

**Fig. 2:**
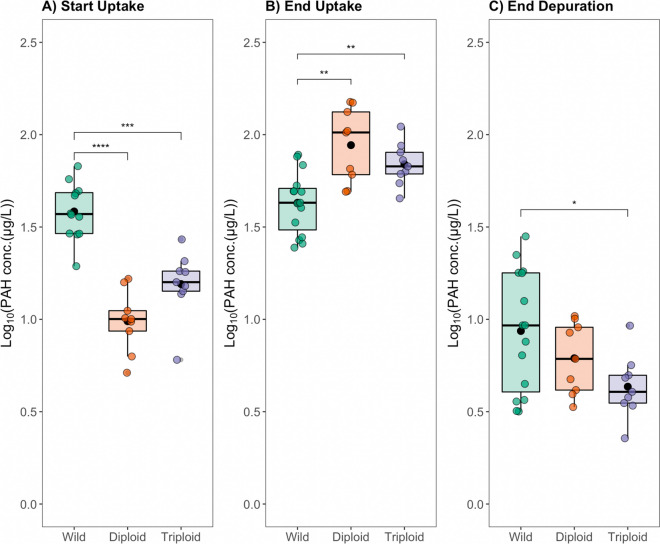
Mean total PAH concentrations in oyster interstitial fluid as measured by antibody-based biosensor technology compared between wild oysters from the Republic site, transplanted diploid oysters, and transplanted triploid oysters at each time point: **A** start of uptake phase (day 0); **B** end of uptake phase (day 30); and **C** end of depuration phase (day 44). During a 30-day uptake period, all oysters were held in cages at a PAH-impacted field site, Republic, in the Elizabeth River (VA, USA). During a 14-day depuration period, oysters were held in the York River (VA, USA). Results were not compared across time points due to an inability to meet the independent data assumption across time for analysis of variance (ANOVA). Brackets with asterisks depict significant results of Games-Howell post hoc comparison, conducted following a significant one-way ANOVA at each respective time point. The ends of each bracket depict which groups were compared and found to be significantly different (e.g., wild vs. triploid in **C**). The number of asterisks above each bracket depicts the cut-off value for each level of significance and is coded as follows: ****0.0001; ***0.001; **0.01, *0.05. Non-significant results (i.e., *p*-value > 0.05) are not shown. For interpretation of the boxplot: The black point depicts the mean, the filled area of the boxplot depicts the interquartile range, with the solid black line depicting the median. Vertical lines (i.e., whiskers) extending from the box depict the upper and lower quartiles

Oysters exhibited significant differences in their uptake and depuration of 3–5 ring PAH concentrations in the oyster interstitial fluid based on their origin of location: wild oysters inhabiting a PAH-impacted field site in the Elizabeth River (the former site of Republic creosote plant), transplanted diploid oysters, and transplanted triploid oysters. At the start of uptake (Fig. 2A), wild oysters had significantly higher concentrations measured in oyster interstitial fluid relative to the transplanted groups. There was no significant difference between transplant concentrations in diploid and triploid cultured oysters. Notably, concentrations above the detection limit were present in both transplanted groups, even though they were originally acquired from aquaculture facilities. At the end of uptake (Fig. 2B), both transplanted groups had accumulated concentrations that were significantly higher than what was measured in the wild oysters. There was no significant difference observed between the two transplanted groups. By the end of depuration in the York River (Fig. 2C), the concentration of residual PAH in oyster interstitial fluid was significantly different between the wild Republic oysters and transplanted triploids. In addition, the variance was much wider in the concentrations found in wild oysters from the contaminated site.

PAH concentrations measured in wild oysters were similar between the start of uptake and end of uptake, supporting the assumption that wild oysters are at steady state with the environment at Republic. The similar levels of PAH accumulated by the end of uptake observed in the transplanted oyster groups align with the results observed in Miles et al. (2014). When transplanted triploid oysters were compared to transplanted diploid oysters that had not yet spawned, similar PAH concentrations were observed. We also found that concentrations were similar between transplanted triploids and diploids suggesting the diploids were in a pre-spawning state during this experiment. Muñoz Sevilla et al. (2017) also observed similar metal concentrations between ploidies collected from three oyster farms throughout a year-long study. However, Miles and colleagues observed significant differences during spawning season in which triploids had overall higher levels relative to diploids. A higher triploid body burden was also observed in a study comparing trace metal bioaccumulation between groups when deployed in an estuary (Robinson et al., 2005).

At the end of depuration, the observation of elevated, though variable, concentrations in the wild Republic oysters relative to the transplanted groups supports findings by Sericano et al. (1996) in which newly exposed transplanted oysters were compared to chronically exposed native oysters inhabiting a PAH-impacted site in the Houston Ship Channel. By the end of depuration, native oysters retained higher levels of PAH relative to the transplanted oysters. Notably, the matrices used for analysis are different between studies so only a general trend comparison can be made. The variability in concentrations measured in wild oysters may be attributed to limitations of passive biomonitoring approaches: an inability to control for biological parameters in wild populations (e.g., sex and life stage) and the requirement of convenience sampling due to the heterogenous distribution of oysters at a site (Beyer et al., 2017).

Mean shell height of the transplanted diploid oysters was significantly longer than both the wild and transplanted triploid groups (Fig. S1). However, when total extractable lipid on a dry weight basis was compared across groups, transplanted triploids had a significantly higher percentage of lipid relative to the wild oysters (Fig. S2). The size difference observed between oysters may be a confounding factor in this study; however, based on the comparison of the lipid per dry weight, diploid size difference may not be an influential factor.

**Fig. 3:**
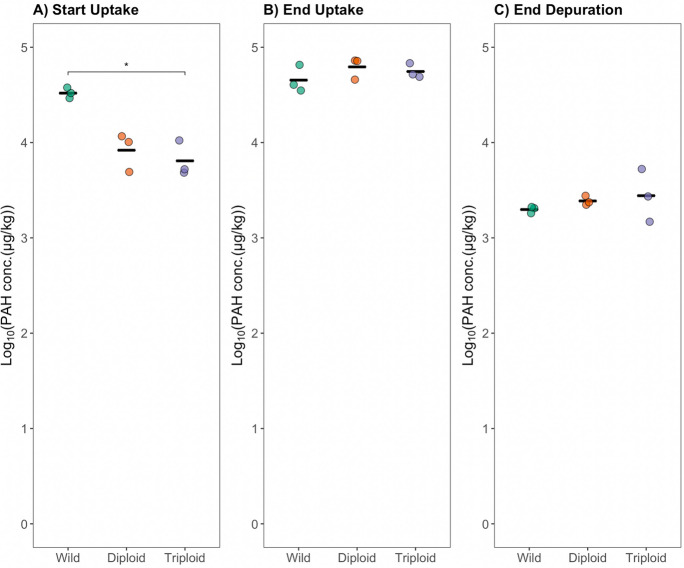
Mean total PAH concentrations in oyster soft tissue measured by GC–MS were compared between wild oysters inhabiting Republic, transplanted diploid oysters, and transplanted triploid oysters at each time point: **A** start of uptake phase (day 0); **B** end of uptake phase (day 30); and **C** end of depuration phase (day 44). During a 30-day uptake period, all oysters were held in cages at a PAH-impacted field site, Republic, in the Elizabeth River (VA, USA). During a 14-day depuration period, oysters were held in the York River (VA, USA). Results were not compared across time point due to an inability to meet the independent data assumption across time for analysis of variance (ANOVA). The black crossbar depicts the mean. Brackets with asterisks depict significant results of Games-Howell post hoc comparison, conducted following a significant one-way ANOVA at each respective time point. The ends of each bracket depict which groups were compared and found to be significantly different (e.g., wild vs. triploid in **C**). The number of asterisks above each bracket depicts the cut-off value for each level of significance and is coded as follows: *0.05. Non-significant results (i.e., *p*-value > 0.05) are not shown

When mean PAH concentrations in oyster tissue were compared between groups at each time point, different trends were observed relative to the oyster interstitial fluid. A significant difference was only observed between wild and transplanted triploids at the start of uptake (Fig. 3A). The wild and transplanted diploids comparison was on the margin of significance (*p* = 0.053). Concentrations were not significantly different between groups at the end of uptake (Fig. 3B) and at the end of depuration (Fig. 3C). Total concentrations per cage were also compared at each time point and no significant difference was detected, suggesting cage assignment did not contribute additional variability (Fig. S3).

The difference in trends observed in tissue concentrations relative to those of oyster interstitial fluid concentrations supports the hypothesis that 30 days was not enough time for the transplanted oysters to achieve steady state with the environment, as has been suggested previously (Schøyen et al., 2017; Sericano et al., 1996). And as an extension, revealed through biosensor analysis of the oyster interstitial fluid, the observed differences in trends between the aqueous (oyster interstitial fluid) and tissue phase within an oyster reflect non-steady state conditions within the oyster as well. The biosensor demonstrates value in facilitating further scrutiny of mechanistic differences in contaminant between transplanted and native groups with its ability to work within small spatiotemporal scales.

### Internal partitioning

#### Digestive gland and gill fluid comparison via biosensor

**Fig. 4:**
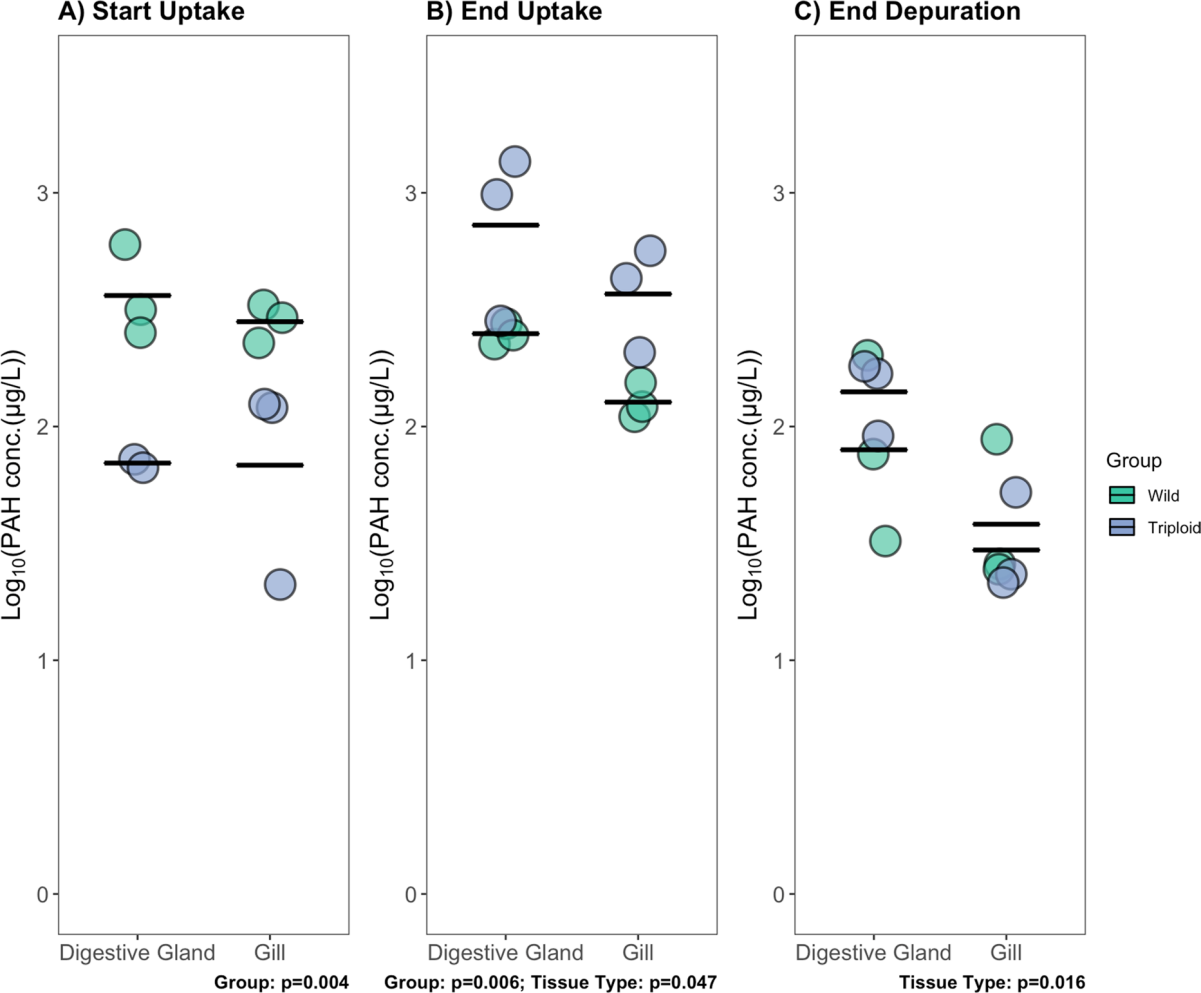
Total 3–5 ring PAH concentrations measured in the digestive gland fluid and gill fluid. Concentrations measured in respective fluid of wild oysters inhabiting a PAH-impacted field site, Republic, in the Elizabeth River (VA, USA) are compared to those of transplanted culture triploid oysters at each sampling point: **A** start of uptake phase (day 0); **B** end of uptake phase (day 30); **C** end of depuration (day 44). During the uptake phase, oysters were held at Republic for a 30-day period. At the end of uptake, all oysters were relocated to the York River (VA, USA) for a 14-day depuration period. Log_10_-transformed individual measurements are shown. The black crossbar depicts the mean for each tissue type for respective groups. Independent two-way ANOVAs were conducted at each time point. Significant *p*-value results are reported below each figure. A 0.05 significance level was selected for analysis. No significant difference between tissue types as a function of oyster group was observed

Overall, differences in PAH concentrations were observed between groups and between fluid types throughout the experiment. At the start of the uptake period (Fig. 4A), wild Republic oysters had a higher level of PAH relative to the transplanted triploid for both fluid types. By the end of uptake (Fig. 4B), transplanted triploids had accumulated a higher concentration of PAH for both fluid types relative to the wild oysters. In addition, when the concentrations between tissue types were compared, a significantly higher level of PAH was measured in the digestive gland fluid relative to gill fluid. At the end of depuration (Fig. 4C), the concentrations between groups were similar; however, the digestive gland fluid had a higher level of residual PAH relative to the gill fluid.

The differences observed in PAH concentrations between groups and between fluid types at each time point suggest that internal partitioning is important in driving the overall PAH concentrations observed in wild and transplanted oysters. At the start of uptake, the concentrations measured in each fluid type for each group are similar, which indicates that internal compartments are at steady state for each oyster group at the start of the experiment. By the end of uptake, a higher level of PAH was measured in the digestive gland. This trend is maintained through the end of the experiment where the digestive gland fluid still retains a significantly higher PAH concentration relative to the gill fluid. These findings provide further evidence that elimination of PAH from this lipid-rich organ is rate-limiting, also supporting previous hypotheses that mechanisms controlling uptake and depuration in bivalves are complex, and that simple one-compartment kinetic models ascribed to these organisms for simplicity may be inappropriate (Beyer et al., 2017; Grech et al., 2017; van Haren & Kooijman, 1993; van Haren et al., 1994). Higher PAH concentrations in bivalve digestive gland tissue relative to other tissue types and slower elimination rates have been measured in previous PAH kinetic studies exploring internal partitioning, indicating that this organ is an important storage site for PAH accumulation (Bustamante et al., 2012; Wang et al., 2020; Widdows et al., 1983). Furthermore, Lacroix et al. (2015) observed higher PAH concentrations in the digestive gland tissue of transplanted mussels relative to wild mussels at an impacted site in a study comparing active and passive biomonitoring methods. With a low-volume sample requirement, antibody-based biosensor technology demonstrates its value in exploring internal partitioning as final digestive gland and gill fluid volumes successfully collected for analysis were typically less than 1 mL. This technology shows promise in facilitating future investigations of mechanisms driving internal partitioning within different compartments in an individual oyster and calibrating ascribed kinetic models. An improved understanding internal chemical dynamics may lead to a better understanding of the differences observed between transplanted and wild groups.

#### IHC-facilitated visualization of PAH in tissue and comparative analysis

**Fig. 5:**
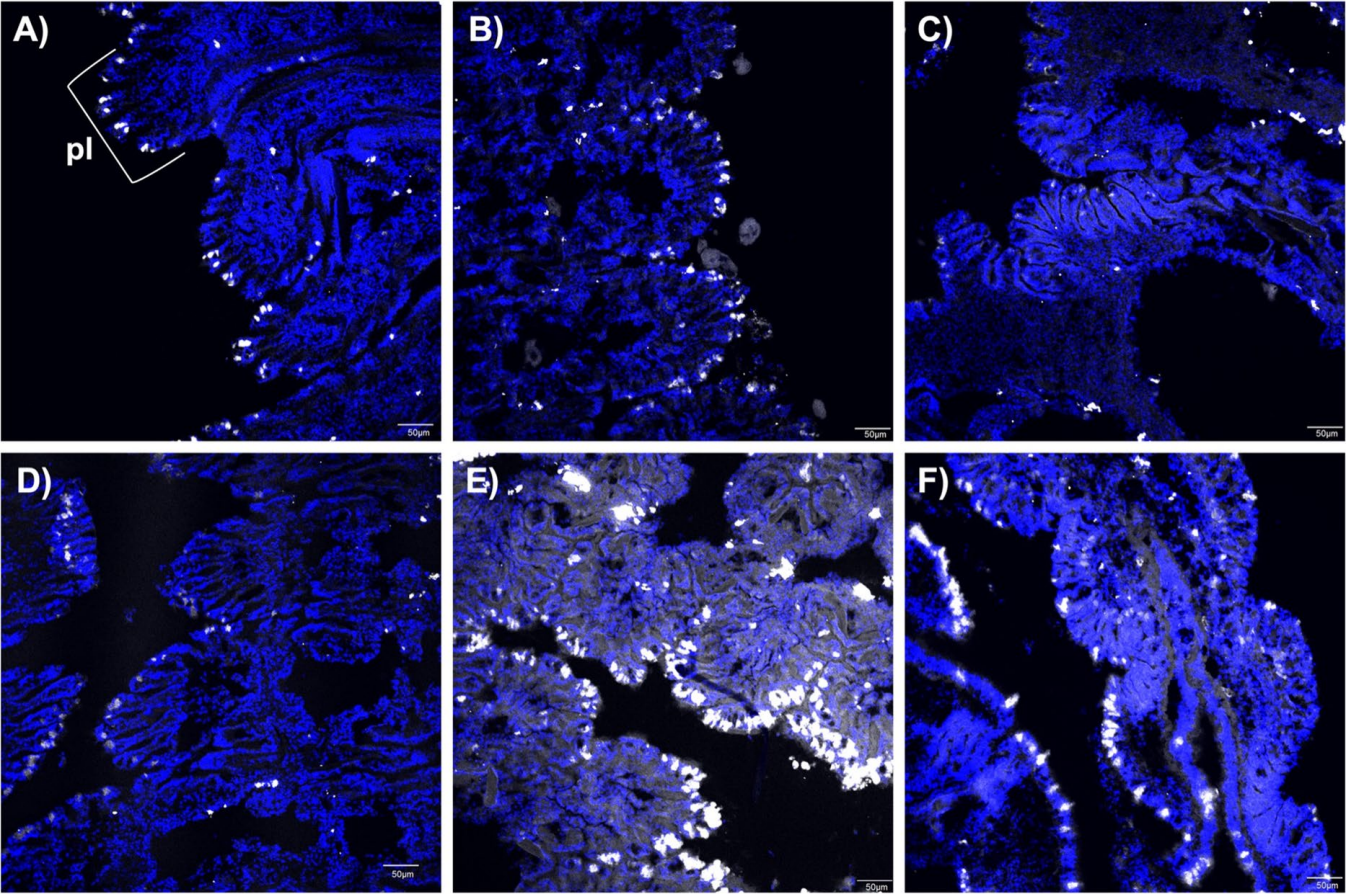
Positive control confocal microscope images of oyster gill tissue collected at each timepoint of the study. Oysters were held at PAH-impacted site in the Elizabeth River for uptake (30 days) and relocated to the York River for 14-day depuration period. **A**–**C** Wild Republic oyster gill at **A** start of uptake (day 0); **B** end of uptake (day 30); **C** end of depuration (day 44). **D**–**F** Transplanted triploid oyster gill at **D** start of uptake; **E** end of uptake; **F** end of depuration. AF647-tagged anti-PAH antibody (mAb 2G8) is depicted in white; 4′,6-diamidino-2-phenylindole (DAPI) stain for cell nuclei depicted in blue; pl, gill plica, the target gill structure for analysis, shown in 5.A as reference

**Fig. 6:**
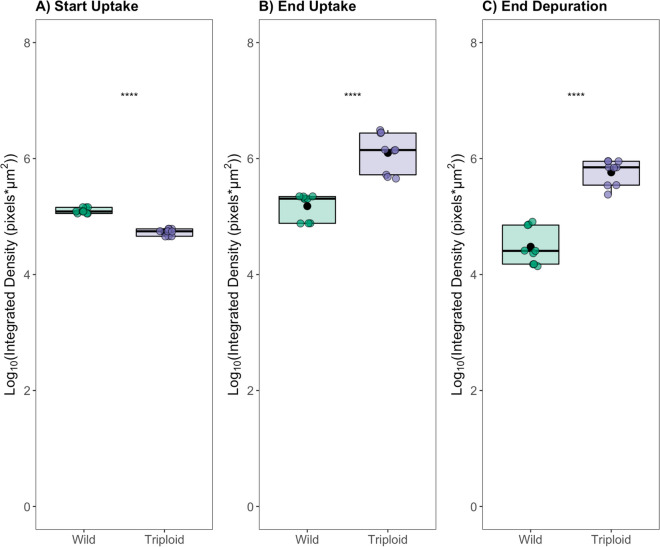
Analysis of the integrated density of mAb 2G8 signal measured in the gill plicae of wild and transplanted triploid oyster as measured from confocal images (Fig. 5). Three plicae per image were measured in triplicate. To satisfy assumptions for statistical analysis, measurements were log_10_-transformed and individual Welch two-sample *t*-tests were performed at each time point: **A** start of uptake phase (day 0); **B** end of uptake phase (day 30); **C** end of depuration (day 44). During the uptake phase, oysters were held at Republic for a 30-day period. At the end of uptake, all oysters were relocated to the York River (VA, USA) for a 14-day depuration period. Asterisks denote a significant difference (*p*-value < 0.05) between group means

IHC analysis provided further visual evidence of differences in internal partitioning between wild and transplanted triploids and these findings were confirmed via a comparative analysis of integrated signal density. The level of signal observed in wild gill tissue did not appear to significantly change between the start and end of the uptake phase at Republic, further confirming steady-state conditions within these oysters (Fig. 5A–B). When transferred to the York River, the level of signal observed in the wild Republic gill was reduced (Fig. 5C). Comparing the intensity of signal observed in wild Republic gill to transplant triploid gill, the signal appears less intense in the transplanted triploids at the start of the uptake phase (Fig. 5D). However, by the end of uptake, the transplanted triploid gill had accumulated a PAH level that far surpassed the level observed in wild Republic gill collected at the same time point as evidenced by the significantly more intense signal (Fig. 5E). By the end of depuration, a higher signal is observed in the transplant triploid gill relative to the wild Republic oyster suggesting higher levels of PAH were retained (Fig. 5F). Negative controls were prepared and autofluorescence was not observed in gill images, confirming that our observed signal is attributed to mAb 2G8 binding to PAH (Fig. S4). Our visual observations at each time point were confirmed when integrated density was measured for three plicae per image and means were compared at each time point (Welch two-sample *t*-test) (Fig. 6).

At the start of uptake, the results of our gill plicae image analysis align with visual observations in Fig. 4A: wild Republic oysters had higher PAH level in gill fluid relative to the transplanted triploids (although not found to be statistically significant). In addition, at the end of uptake, the results of gill image analysis align with our visual comparisons in Fig. 4B that transplanted triploids had accumulated PAH in gill fluid that surpassed the levels measured in wild oyster gill fluid (although determined to be statistically insignificant). The trend observed at the end of depuration in image analysis differed from our findings in gill fluid concentration comparison at the same time point Fig. 4C in which concentrations were similar. It is important to note the small sample size for tissue fluid comparison and immunohistochemical comparison. Additionally, interference due to suspected autofluorescence prevented an adequate visual comparison of PAH binding and further analysis of digestive gland images (Fig. S5 and S6). However, our findings suggest the incorporation of immunohistochemical visualization in analysis could be valuable in understanding PAH kinetics in organisms and possible mechanistic differences between wild and transplanted groups. Immunohistochemical visualization utilizing mAb 2G8 could be coupled with biomarker studies investigating potential metabolic adaptations in chronically exposed wild population that may reduce stress response.

## Conclusions

Using novel immunological techniques and conventional GC–MS analysis, we explored differences in PAH concentrations between wild oysters inhabiting a PAH-impacted field site in the Elizabeth River, Republic, and cultured triploid and diploid oysters. We also preliminarily investigated differences in internal partitioning between wild Republic oysters and transplanted triploid oysters. These findings support the hypothesis that kinetic rates and insufficient equilibration time (both with the environment and within internal phases of the oyster) may significantly contribute to observed differences in wild and transplanted groups. Future work should incorporate seasonal variability to observe differences in PAH body burden and energy budgeting between triploid and diploid oysters post-spawning. Dynamic energy budgeting models have been previously employed to account for biological factors, such as physiology, energy storage, and reproductive status, that can influence contaminant uptake (van Haren et al., 1994; van Haren & Koojman, 1993; Grech et al., 2017; Beyer et al., 2017). More work is needed to better understand how a difference in energy budgeting and reproductive status in triploids could impact overall contaminant body burden. This is important not only for further evaluation of triploid oysters as a novel biomonitoring tool, but for understanding potential adverse health risks associated with consuming this popular seafood product.

To explore the differences in internal partitioning within an oyster, digestive gland homogenate fluid and gill homogenate fluid were compared between wild Republic oysters and transplanted triploids. Although a significant difference in concentration was not detected between the different tissue-specific fluids of respective groups, overall differences were observed between tissue types and between treatment groups. At the start of uptake, the wild Republic oysters had higher concentration overall relative to transplanted triploids; at the end of uptake, transplanted triploids had accumulated a higher overall concertation relative to what was measured in the wild oysters. This aligns with the trends observed in the oyster interstitial fluid at this time point. The digestive gland fluid concentrations were significantly higher than gill fluid concentrations at the end of uptake and end of depuration, suggesting the importance of the digestive gland in sequestering PAH. Overall, our findings suggest that internal partitioning may be an important factor in total concentrations measured in an oyster. Visualization of PAH accumulation in oyster gill tissue and subsequent image analysis supports our observed trends at the start and end of uptake in overall concentration differences between groups.

The decision of which biomonitoring method to use may depend on the research question and objectives. Passive biomonitoring may be more valuable for long-term monitoring of impacted sites and remediation progress. To evaluate an acute exposure, active biomonitoring may be the better approach. However, it is still necessary to have a better understanding of why differences in total PAH concentrations are observed between methods. By applying of sensitive analytical approaches such as antibody-based biosensor instrumentation and immunohistochemistry, we demonstrate their value in investigating mechanisms driving PAH kinetics in oysters. The low-volume sample requirement of biosensor technology allows for the analysis of individual oysters and fluid samples from individual organs. These features make investigating small-scope mechanistic questions much more feasible. With the employment of mAb 2G8, selective for a range of 3–5 PAH compounds, in an immunohistochemical application, we can visualize bioaccumulation of a PAH mixtures to which oysters were exposed in the environment. Although our exploration into internal partitioning differences between transplanted triploids and wild oysters is preliminary in nature, nevertheless, we further demonstrate the value of incorporating a novel approach for addressing environmental chemistry-related questions that cannot be as sufficiently answered by conventional methods. This includes understanding the differences between active and passive biomonitoring methods.

### Supplementary Information

Below is the link to the electronic supplementary material.

Supplementary file1 (DOCX 13262 KB)

## Data Availability

Data are available via the corresponding author upon request.
