# Supplementary material for: Exploring PAH kinetics in wild vs. transplanted triploid and diploid oysters at a contaminated field site using immunological techniques
Source: Environ Monit Assess. 2023 Nov 13;195(12):1462. doi: 10.1007/s10661-023-12064-1 (PMC10643322; doi:10.1007/s10661-023-12064-1)
Supplement: Supplementary file 1 — Supplementary file1 (DOCX 13262 KB) [file 10661_2023_12064_MOESM1_ESM.docx]

**Supplementary Information**

Exploring PAH Kinetics in Transplanted Triploid and Diploid vs. Wild Oysters at an Impacted Field Site Using Immunological Techniques


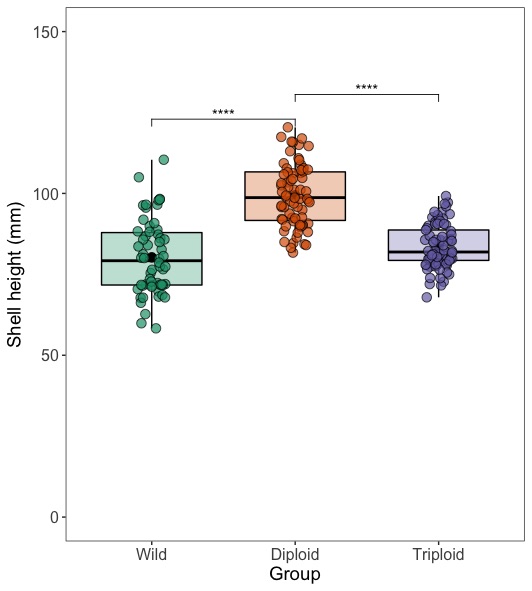


**Figure S1.** Shell height (mm) were measured by calipers and compared between wild oysters inhabiting Republic, transplanted diploid oysters, and transplanted triploid oysters. The black point depicts the mean. Brackets with asterisks depict significant results of Games-Howell post hoc comparison, conducted following a significant one-way ANOVA at each respective time point (p-value < 0.05). Non-significant results (p-value > 0.05) are not shown. For interpretation of the boxplot: the filled area of the boxplot depicts the interquartile range, with the solid black line depicting the median. Vertical lines (i.e. whiskers) extending from the box depict the upper and lower quartiles.


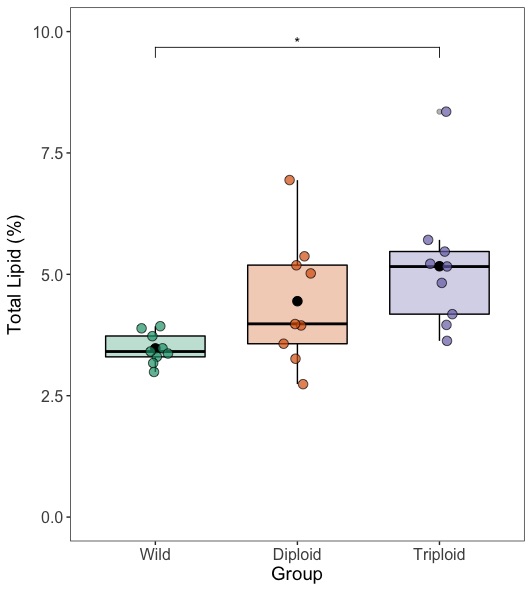


**Figure S2.** Total extractable lipid content was measured on a dry weight basis following HPLC-FLD. Lipid content was compared between wild oysters inhabiting Republic, transplanted diploid oysters, and transplanted triploid oysters. The black point depicts the mean. Brackets with asterisks depict significant results of Games-Howell post hoc comparison, conducted following a significant one-way ANOVA at each respective time point (p-value < 0.05). Non-significant results (i.e. p-value > 0.05) are not shown. For interpretation of the boxplot: the filled area of the boxplot depicts the interquartile range, with the solid black line depicting the median. Vertical lines (i.e. whiskers) extending from the box depict the upper and lower quartiles.


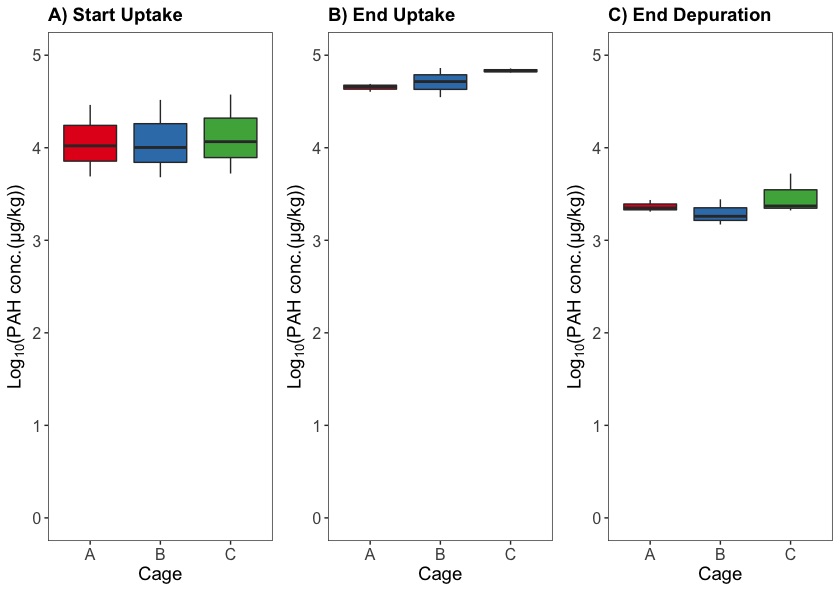


**Figure S3.** Comparison of log_10_-transformed tissue concentrations between cages to assess potential cage effect at each time point: **A)** start of uptake phase (Day 0); **B)** end of uptake phase (Day 30); and **C)** end of depuration phase (Day 44). For GC–MS analysis, 3-5 oysters per cage were pooled to create a homogenized composite sample. Independent one-way ANOVAs at each time point determined that there were no significant differences in concentrations due to cage assignment.


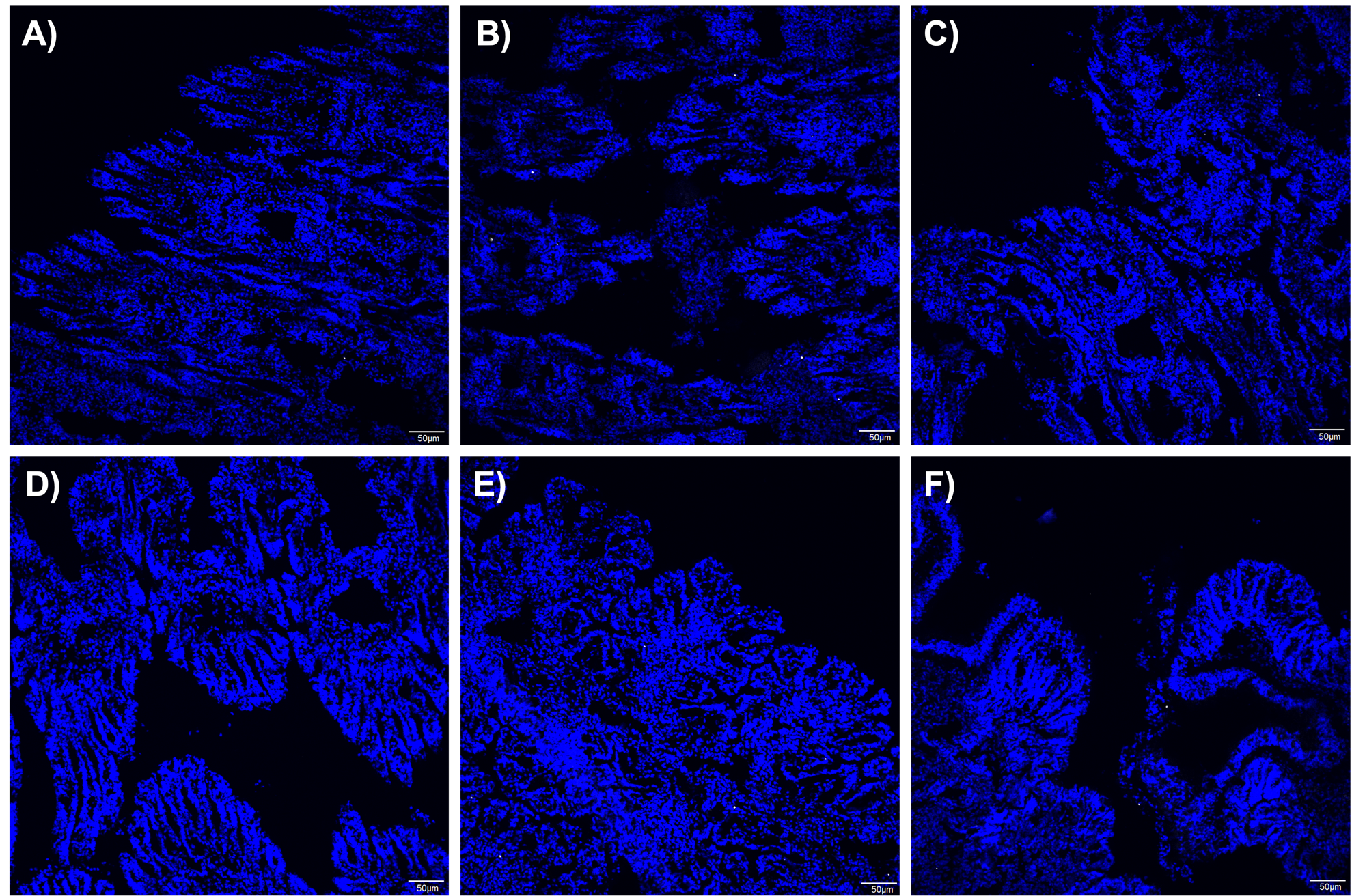


**Figure S4.** Negative control confocal microscope images of oyster gill tissue collected at each timepoint of the study. Oysters were held at PAH-impacted site in the Elizabeth River for uptake (30 days) and relocated to the York River for 14-day depuration period. **S4.A-C)** wild Republic oyster gill at **S4.A)** start of uptake (Day 0); **S4.B)** end of uptake (Day 30); **S4.C)** end of depuration (Day 44). **S4.D-F)** transplanted triploid oyster gill at **S4.D)** start of uptake; **S4.E)** end of uptake; **S4.F)** end of depuration. 4′,6-diamidino-2-phenylindole (DAPI) stain for cell nuclei depicted in blue; pl=gill plica, the target gill structure for throughout analysis, shown in S4A as reference.


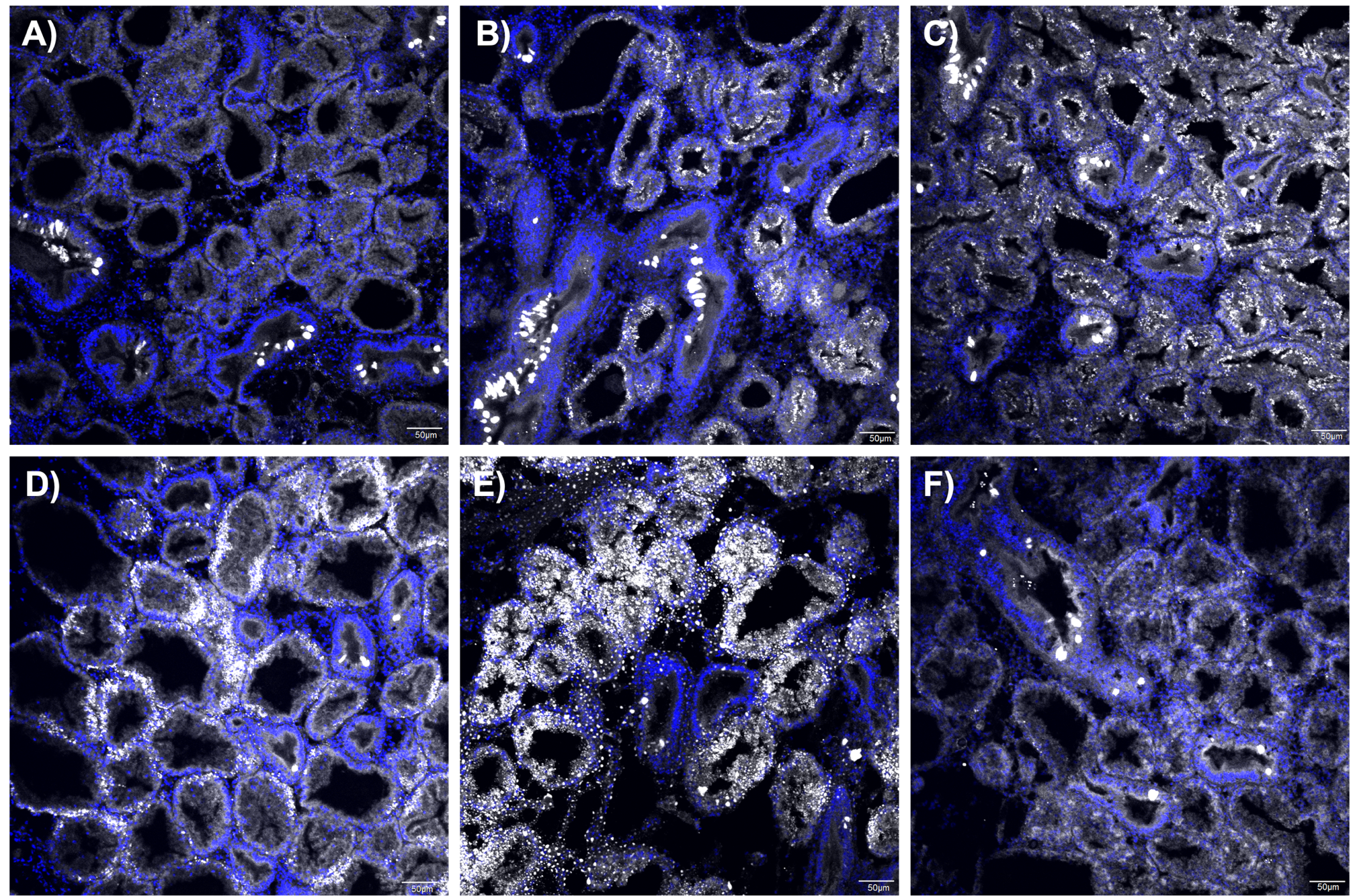


**Figure S5.** Positive control confocal microscope images of oyster digestive gland tissue collected at each timepoint of the study. Oysters were held at PAH-impacted site in the Elizabeth River for uptake (30 days) and relocated to the York River for 14-day depuration period. **S5.A-C)** wild Republic oyster digestive gland at **S5.A)** start of uptake (Day 0); **S5.B)** end of uptake (Day 30); **S5.C)** end of depuration (Day 44). **S5.D-F)** transplanted triploid oyster digestive gland at **S5.D)** start of uptake; **S5.E)** end of uptake; **S5.F)** end of depuration. AF647-tagged anti-PAH antibody (mAb 2G8) is depicted in white (however, autofluorescence is detected in the same wavelength, see Supplemental Figure S6.A-F); 4′,6-diamidino-2-phenylindole (DAPI) stain for cell nuclei depicted in blue.


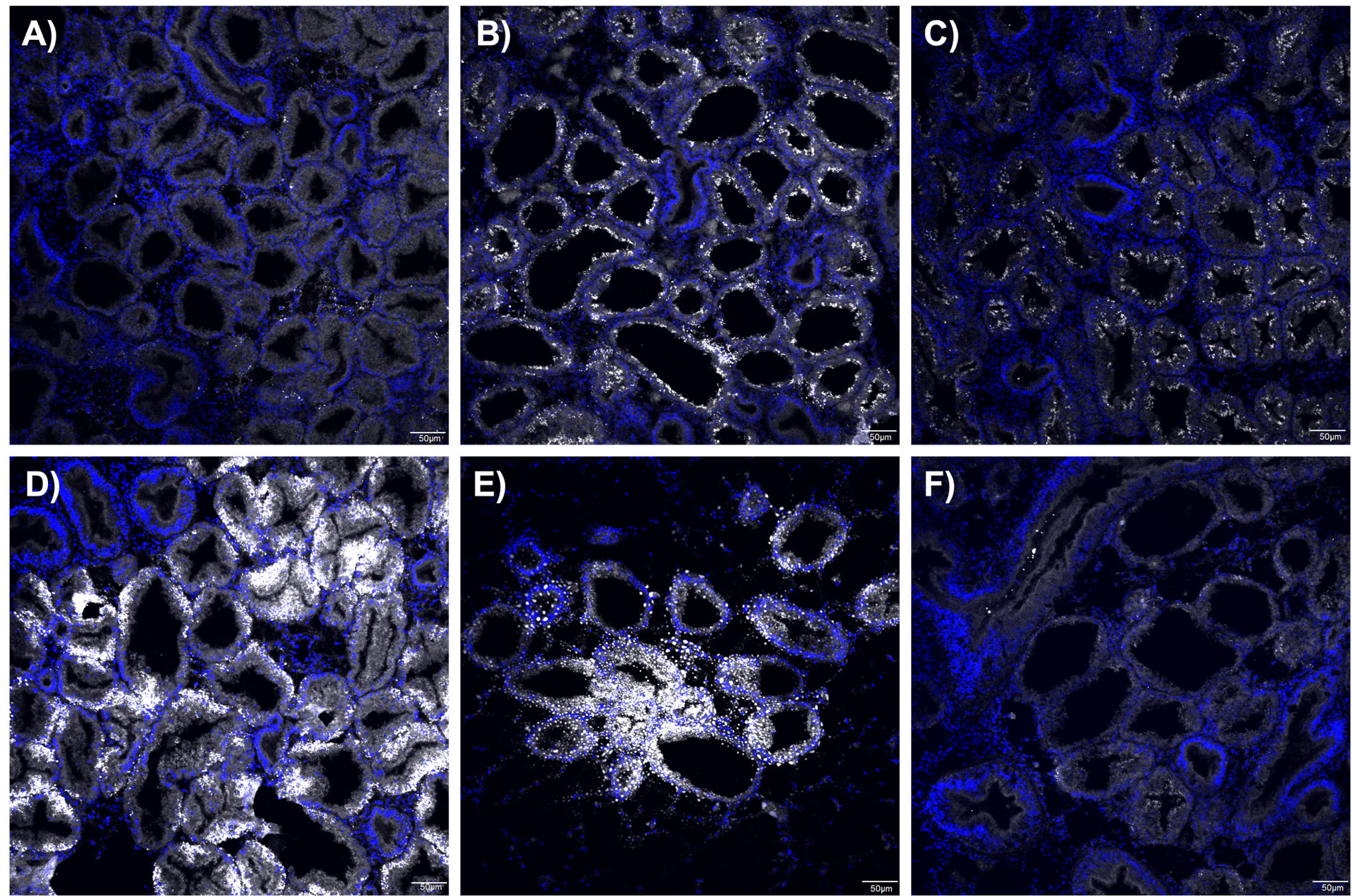


**Figure S6.** Negative control confocal microscope images of oyster digestive gland tissue collected at each timepoint of the study. Oysters were held at PAH-impacted site in the Elizabeth River for uptake (30 days) and relocated to the York River for 14-day depuration period. **S6.A-C)** wild Republic oyster digestive gland at **S6.A)** start of uptake (Day 0); **S6.B)** end of uptake (Day 30); **S6.C)** end of depuration (Day 44). **S6.D-F)** transplanted triploid oyster digestive gland at **S6.D)** start of uptake; **S6.E)** end of uptake; **S6.F)** end of depuration. Suspected autofluorescence (white) is detected in the same wavelength, as AF647-tagged mAB 2G8 see Supplemental Figure S5.A-F); 4′,6-diamidino-2-phenylindole (DAPI) stain for cell nuclei depicted in blue.

Report of ANOVAs and Pairwise comparisons conducted in R

# STAT.2: *One-Way ANOVAs for comparison oyster interstitial fluid concentration (measured via biosensor) between groups at each time point* *and post hoc comparisons reported in Figure 2*

##

## A) Start of Uptake (Day 0)

T0.anova

## ANOVA Table (type II tests)
##
## Effect DFn DFd F p p<.05 ges
## 1 Group 2 27 35.918 2.47e-08 * 0.727

## PAIRWISE COMPARISON (Games-Howell post hoc test)

pwc_T0

## # A tibble: 3 x 8
## .y. group1 group2 estimate conf.low conf.high p.adj p.adj.signif
## * <chr> <chr> <chr> <dbl> <dbl> <dbl> <dbl> <chr>
## 1 conc_log10 Wild Diploid -0.594 -0.775 -0.413 0.000000612 ****
## 2 conc_log10 Wild Triploid -0.393 -0.584 -0.202 0.000211 ***
## 3 conc_log10 Diploid Triploid 0.201 -0.00825 0.411 0.061 ns

##

## B) End of Uptake (Day 30)

T4.anova

## ANOVA Table (type II tests)
##
## Effect DFn DFd F p p<.05 ges
## 1 Group 2 30 11.137 0.000241 * 0.426

## PAIRWISE COMPARISON (Games-Howell post hoc test)

pwc_T4

## # A tibble: 3 x 8
## .y. group1 group2 estimate conf.low conf.high p.adj p.adj.signif
## * <chr> <chr> <chr> <dbl> <dbl> <dbl> <dbl> <chr>
## 1 conc_log10 Wild Diploid 0.312 0.106 0.518 0.004 **
## 2 conc_log10 Wild Triploid 0.208 0.0636 0.353 0.004 **
## 3 conc_log10 Diploid Triploid -0.104 -0.306 0.0994 0.394 ns

##

## C) End of Depuration (Day 44)

T6.anova

## ANOVA Table (type II tests)
##
## Effect DFn DFd F p p<.05 ges
## 1 Group 2 30 3.79 0.034 * 0.202

## PAIRWISE COMPARISON (Games-Howell post hoc test)

pwc_T6

## # A tibble: 3 x 8
## .y. group1 group2 estimate conf.low conf.high p.adj p.adj.signif
## * <chr> <chr> <chr> <dbl> <dbl> <dbl> <dbl> <chr>
## 1 conc_log10 Wild Diploid -0.147 -0.416 0.121 0.367 ns
## 2 conc_log10 Wild Triploid -0.301 -0.559 -0.0442 0.02 *
## 3 conc_log10 Diploid Triploid -0.154 -0.375 0.0668 0.201 ns

#

# STAT.3*: One-Way ANOVAs for comparison oyster tissue concentration (measured via GC–MS) between groups at each time point and pairwise comparisons reported in Figure 3*

## A) Start of Uptake (Day 0)

GCT0.anova

## ANOVA Table (type II tests)
##
## Effect DFn DFd F p p<.05 ges
## 1 Group 2 6 16.829 0.003 * 0.849

## PAIRWISE COMPARISON (Games-Howell post hoc test)

GCpwc_T0

## # A tibble: 3 x 8
## .y. group1 group2 estimate conf.low conf.high p.adj p.adj.signif
## * <chr> <chr> <chr> <dbl> <dbl> <dbl> <dbl> <chr>
## 1 conc_log10 Wild Diploid -0.599 -1.22 0.0180 0.053 ns
## 2 conc_log10 Wild Triploid -0.710 -1.27 -0.150 0.03 *
## 3 conc_log10 Diploid Triploid -0.112 -0.676 0.453 0.773 ns

##

## B) End of Uptake (Day 30)

GCT4.anova

## ANOVA Table (type II tests)
##
## Effect DFn DFd F p p<.05 ges
## 1 Group 2 6 1.165 0.374 0.28

##

## C) End of Depuration (Day 44)

GCT6.anova

## ANOVA Table (type II tests)
##
## Effect DFn DFd F p p<.05 ges
## 1 Group 2 6 0.609 0.574 0.169

#

# STAT.4: *Two-Way ANOVAs for comparison of oyster gill and digestive gland fluid concentrations (via biosensor) between wild Republic oysters and transplanted triploid oysters reported in Figure 4*

## A) Start of Uptake (Day 0)

IP.anova_T0

## ANOVA Table (type II tests)
##
## Effect DFn DFd F p p<.05 ges
## 1 Group 1 7 17.066 0.004 * 0.709
## 2 Type 1 7 0.172 0.690 0.024
## 3 Group:Type 1 7 0.103 0.758 0.014

##

## B) End of Uptake (Day 30)

IP.anova_T4

## ANOVA Table (type II tests)
##
## Effect DFn DFd F p p<.05 ges
## 1 Group 1 8 13.7590000 0.006 * 6.32e-01
## 2 Type 1 8 5.5240000 0.047 * 4.08e-01
## 3 Group:Type 1 8 0.0000445 0.995 5.57e-06

##

## C) End of Depuration (Day 44)

IP.anova_T6

## ANOVA Table (type II tests)
##
## Effect DFn DFd F p p<.05 ges
## 1 Group 1 8 0.175 0.687 0.021
## 2 Type 1 8 9.163 0.016 * 0.534
## 3 Group:Type 1 8 1.189 0.307 0.129
